# Combined Salt and Heat Stress Aggravates Oxidative Stress and Photosynthetic Damage, Disrupting Carbon and Nitrogen Metabolism and Yield in Rice

**DOI:** 10.3390/antiox15030308

**Published:** 2026-02-28

**Authors:** Lin Li, Jie Xu, Jinqi Liu, Wenhao Bi, Yingjiang Li, Aibin He, Xiayu Guo, Zhiyong Ai

**Affiliations:** 1 National Center of Technology Innovation for Saline-Alkali Tolerant Rice in Sanya, Sanya 572024, China; lilin18@hainanu.edu.cn (L.L.); xujie11272026@126.com (J.X.); 13755161912@163.com (Y.L.); heaibing@hhrrc.ac.cn (A.H.); 2 College of Breeding and Multiplication (Sanya Institute of Breeding and Multiplication), Hainan University, Sanya 572025, China; 3 State Key Laboratory of Hybrid Rice, Hunan Hybrid Rice Research Center, Changsha 410125, China

**Keywords:** antioxidants, stress, photosynthesis, non-structural carbohydrates, panicle development

## Abstract

In the context of global climate change, the co-occurrence of salt and heat stress represents a major constraint to rice production, resulting in greater yield penalties than either stress alone. This study aimed to assess the effects of salt and heat stress on oxidative homeostasis, photosynthetic performance, carbon (C)–nitrogen (N) metabolism, and rice yield. The experiment comprised four treatments, i.e., control (CK), salt (irrigation with 3.9 dS m^−1^ NaCl solution), heat (exposure to 36 °C/30 °C day/night for 5 days at panicle initiation), and combined salt + heat stress. Results showed that combined stress enhanced reactive oxygen species (ROS) accumulation (i.e., H_2_O_2_ content and O_2_^−^ contents were 1.3 and 1.5 times higher than CK), and the activities of superoxide dismutase (SOD), peroxidase (POD), and catalase (CAT) were increased by 64.6%, 69.5%, and 74.8% higher than CK. At the molecular level, salt + heat stress upregulated antioxidant defense-related genes, i.e., *OsAPX2*, *OsSODCC1*, and *OsAPX1*, while significantly downregulated ion homeostasis-related genes, i.e., *OsSOSs*, *OsHKT1;3*, *OsHKT1;5*, and *OsNHX4*, and photosynthesis-related genes, i.e., *Ospsbo*, *OsRbcS2*, and *OsRbcS3*, compared with CK. Furthermore, salt + heat stress reduced the activities of C-metabolism enzymes (sucrose phosphate synthase, sucrose synthase, and starch synthase) and N-metabolism enzymes (nitrate reductase, glutamine synthetase, and glutamate synthase), leading to 34.3% and 18.6% lower stem-sheath non-structural carbohydrate accumulation in stem sheath and its translocation rate, respectively, while total N accumulation decreased by 42.9%, as compared with CK. Ultimately, these cascading effects inhibited panicle development and reduced yield. The findings provide a theoretical basis for improving rice tolerance to combined abiotic stresses by targeting oxidative stress mitigation, photosynthetic protection, and key stress-responsive gene regulation.

## 1. Introduction

Rice (*Oryza sativa* L.) is a staple food crop feeding over half of the global population, and its stable productivity is critical for ensuring global food security [1,2]. With the intensification of global climate change, abiotic stresses have emerged as major constraints on rice production, particularly in key rice-growing regions such as the coastal areas of China [3]. Simultaneous occurrence of salt and heat stress during critical reproductive stages of rice, such as panicle initiation, severely disrupts physiological processes and yield formation [4].

Salt stress primarily acts through osmotic stress and ionic toxicity; excessive sodium ion (Na^+^) accumulation disrupts ion homeostasis in rice cells, damaging cell membranes and increasing electrolyte leakage [5]. It also induces stomatal closure, reduces chlorophyll content, and inhibits photosynthetic carbon fixation, while triggering overaccumulation of reactive oxygen species (ROS) that further exacerbates cellular damage [6]. For instance, salt stress can reduce the net photosynthetic rate (P_n_) in rice and increase hydrogen dioxide (H_2_O_2_) content compared with normal conditions [7]. Heat stress, on the other hand, mainly targets the photosynthetic system and metabolic enzymes. For instance, high temperatures (≥36 °C) denature key photosynthetic enzymes such as Rubisco, disrupt the stability of photosystem II (PSII), and reduce the maximum photochemical efficiency [8]. Additionally, heat stress inhibits antioxidant enzyme activity, leading to ROS burst and membrane lipid peroxidation [9,10]. Previous studies have shown that the critical windows for heat-induced damage in rice are the heading–flowering and panicle initiation stage [11]. While the mechanisms of high-temperature injury and its impacts on spikelet sterility during heading–flowering have been extensively investigated, the research on the impacts of heat stress during the panicle initiation stage in rice remains limited.

Previous studies have shown that salt and heat stress independently damage plant physiological processes, but their combined effects are more complex and destructive [12,13]. The combined stress exerts synergistic or additive destructive effects and causes more severe impairment to membrane stability, photosynthetic rate, and plant biomass than either single stress [3]. The wheat seedlings under combined heat and salt stress show enhanced Na^+^ accumulation, reduced relative water contents, and decreased shoot biomass compared to individual stress [14]. Despite extensive research on the individual effects of salt and heat stress on rice, the mechanisms by which their combined effects on rice physiology remain elusive [15,16]. Notably, existing studies have confirmed that there is significant genetic variability in the response of different rice genotypes to individual and combined salt and heat stress; contrasting tolerant and sensitive genotypes are critical materials for dissecting the metabolic and molecular mechanisms underlying differential stress susceptibility, as well as for mining elite stress-tolerant genes for rice breeding [17,18]. Meanwhile, rice is generally recognized as a moderately salt-tolerant crop, while the breeding of mainstream commercial rice varieties has long been centered on improving yield potential, and the genetic improvement of synergistic tolerance to multiple abiotic stresses (including combined salt and heat stress) has been seriously neglected.

Carbon (C)–nitrogen (N) metabolism serves as the primary energy reserve and carbon source for rice panicle development, and its synthesis and remobilization are strongly linked to photosynthetic efficiency and oxidative balance [17]. ROS overaccumulation inhibits carbon metabolism enzymes (e.g., sucrose synthase, starch hydrolase), while photosynthetic damage reduces nonstructural carbohydrate (NSC) precursor supply [9]. Together, these effects lead to insufficient NSC allocation to panicles, resulting in spikelet abortion and heading delay [18]. However, how combined salt and heat stress disrupts these processes to inhibit panicle development and reduce yield has not been systematically elucidated. Against this background, this study hypothesized that the combined salt and heat stress synergistically aggravates oxidative stress, photosynthetic damage, disrupts C-N metabolism and remobilization, interferes with panicle development, and causes yield loss. To verify this hypothesis and address the above research gaps, the core objectives of this study are: (1) to compare the differential effects of single salt, single heat, and combined salt and heat stress on rice oxidative homeostasis, ion balance, and photosynthetic performance; (2) to elucidate the regulatory mechanism of combined salt and heat stress on key enzyme activities of carbon and nitrogen metabolism, as well as the accumulation and remobilization of non-structural carbohydrates and nitrogen in rice; and (3) to clarify the cascade pathway of combined salt and heat stress leading to rice yield loss, and provide a targeted theoretical basis for the genetic improvement of multi-stress tolerance in commercial rice varieties.

## 2. Materials and Methods

### 2.1. Plant Materials and Growth Conditions

A pot experiment was conducted at the National Center of Technology Innovation for Saline–Alkali Tolerant Rice (18°35′ N, 109°17′ E). Seeds of a widely cultivated rice cultivar ‘Ruanhuayoujinsi’ in South China were surface-sterilized with 1% NaClO for 10 min, rinsed with distilled water, soaked in water at 30 °C for 24 h, and allowed to germinate at 32 °C for 48 h. Germinated seeds were sown in 72-hole seedling trays filled with nutrient soil (peat: vermiculite: perlite = 3:1:1). On 14 July 2026, rice seedlings were transplanted at the three-leaf stage (25 d after sowing), with two seedlings per hill and three hills per pot to ensure transplanting survival rate. At seven days after transplanting (seedling recovery period), standard thinning was performed: only one uniformly growing and robust seedling was retained per hill, resulting in a final total of three rice plants per pot for all treatments throughout the whole growth period (Figure S1). The standard normal growth conditions during this period were based on the measured average meteorological data of the experimental site during the rice growth period: natural day/night photoperiod of 13–14 h light per day, average daily maximum temperature of 29.4 °C, average daily minimum temperature of 25.3 °C, with a daily average temperature of 27.3 °C, average daily total solar radiation of 17.1 MJ m^−2^ (corresponding to an average daily photosynthetic photon flux density of ~729.1 μmol m^−2^ s^−1^, Figure S2), and relative humidity of 60–80%. Based on the confirmed transplanting date of 14 July 2026, key growth stages of rice were clearly defined using days after transplanting (DAT) as the unified time scale throughout the experiment: The full experiment adopted a unified timeline with 14 July 2026 as the transplanting date (0 days after transplanting, DAT). Key growth stages and experimental operations are detailed below: 20 DAT: Salt stress treatment initiated; 49 DAT: 5-day continuous heat stress treatment initiated at the panicle initiation stage; 54 DAT: Heat stress terminated; 73 DAT: Full heading stage; 98 DAT: Physiological maturity stage; plants harvested for yield trait evaluation and final biomass/nutrient accumulation determination.

The pots used were 36 cm (top diameter) × 26 cm (bottom diameter) × 28 cm (height). The soil was air-dried, sieved (2 mm), and filled with 15 kg of mixed soil. The 2.76 g urea per pot (containing 46% N) applied at a ratio of 1:1:1 for basal: tillering: panicle fertilizer; 3.03 g superphosphate (containing 12% P_2_O_5_) applied once as basal fertilizer; and 1.18 g potassium chloride (containing 60% K_2_O) was applied at a 1:1 ratio for basal: panicle fertilizer. This fertilization scheme was based on Li et al. [19] to ensure sufficient nutrient supply and eliminate intraspecific nutrient competition. The normal growth state of rice plants in pots throughout the heading stage is shown in Figure S1. The experimental paddy soil used for potting contained 22.6 g kg^−1^ organic matter, 1.32 g kg^−1^ total N, 45.8 mg kg^−1^ available P, 81.5 mg kg^−1^ available K, with an initial pH of 6.8 and electrical conductivity (EC) of 0.12 dS m^−1^.

### 2.2. Experimental Design

The experiment was arranged in a completely randomized design (CRD) with four treatments: control (CK), salt stress, heat stress, and combined salt + heat stress. A total of 80 pots were used in the experiment, with 20 pots per treatment. For each treatment, the 20 pots were randomly divided into 4 independent groups, with 5 pots per group. Each group of 5 pots was defined as one independent experimental unit, which also served as one independent biological replicate for subsequent index determination and statistical analysis. All pots were randomly placed in the natural field conditions in a rain-proof screen house. To minimize the effects of environmental gradients (e.g., light, temperature, and air flow), all pots were randomly rotated every three days. CK: No salt or heat stress; irrigated with tap water and grown under normal temperature throughout the growth period. Salt stress: Salt stress treatment was initiated at the tillering stage (20 days after transplanting, DAT), by irrigating with NaCl solution with an electrical conductivity (EC) of 3.9 dS m^−1^, to maintain a stable target EC of 4.1 ± 0.2 dS m^−1^ in the root zone (5–10 cm soil depth) throughout the entire growth period. Soil EC was measured in situ directly using a calibrated portable soil conductivity meter (2266FS, Spectrum Technologies, Inc., Aurora, IL, USA). For each pot, three replicate measurement points were randomly selected in the root zone, and the average value was taken as the soil EC of the pot. Monitoring was performed every 3 days; if the measured EC fell below the target range of 4.1 ± 0.2 dS m^−1^, supplementary irrigation with the 3.9 dS m^−1^ NaCl solution was performed immediately to restore and maintain the stable target salinity level [19]. Heat stress: Heat stress treatment was conducted at the panicle initiation stage (49 days after transplanting). The plants grown under normal conditions were transferred to a climate chamber at the panicle initiation stage and exposed to 36 °C/30 °C day/night temperature for 5 days [10]. Combined salt + heat stress: plants were exposed to the combination of salt and heat stress treatments. All potted plants from all treatment groups were synchronously transferred to identical programmable artificial climate chambers to eliminate the interference of environmental heterogeneity. To avoid pseudoreplication and eliminate the interference of environmental gradients within the climate chambers, all pots from all treatment groups were completely randomly arranged in the chambers, and their positions were randomly re-rotated every 2 days throughout the entire stress treatment period. To ensure temperature was the only variable in the experiment, all environmental parameters except the day/night temperature regime were set completely consistent across all chambers and treatment groups, including photosynthetic photon flux density of 1200 μmol m^−2^s^−1^, relative humidity of 75 ± 5%, and a photoperiod of 12 h/12 h (day/night). The temperature regime was set as follows: the CK and salt were maintained at the field’s natural normal temperature regime of 29.35 °C/25.30 °C (day/night). After the heat stress, all potted plants from all treatment groups were moved back to the original natural environment, and grown with conventional management until physiological maturity.

### 2.3. Measurement Indexes and Methods

#### 2.3.1. Oxidative Stress and Antioxidant Enzyme Activity

At the post-heat stress (exposure to 36 °C/30 °C day/night for 5 days at panicle initiation) and full heading stage (about 25 days after panicle initiation), the top fully expanded leaves were collected and stored at –80 °C to estimate ROS accumulation and antioxidant enzyme activity. The H_2_O_2_, O_2_^−^, malondialdehyde (MDA) content, superoxide dismutase (SOD), peroxidase (POD), and catalase (CAT) activities were determined via assay kits provided by Jiangsu Jingmei Biotechnology Co., Ltd. (Yancheng, China). The detailed measurement method was found in Supplementary Material.

#### 2.3.2. Photosynthetic Parameters and Aboveground Biomass

At the post-heat stress and heading stage, photosynthetic parameters of the top fully expanded leaf were measured using a Li-6800XT portable photosynthetic analyzer (LiCor, Lincoln, NE, USA), including P_n_, stomatal conductance (g_s_), intercellular CO_2_ concentration (Cᵢ), and transpiration rate (Tr). At the heading and maturity stages, six representative individual rice plants (hills) were randomly sampled from each treatment. The complete aboveground part of each plant, including the main stem and all effective tillers, was rinsed with distilled water, and then partitioned into stem sheaths, leaves, and panicles.

#### 2.3.3. C and N Metabolism Enzyme Activity

At the post-heat stress and heading stage, the NSC metabolism enzyme activities, such as sucrose phosphate synthase (SPS), sucrose synthase (SS), and starch synthase (SSS), were estimated. Fresh stem-sheath tissue (0.5 g) was determined using the detection kits provided by Jiangsu Jingmei Biotechnology Co., Ltd. The activities of leaf N metabolism enzymes such as nitrate reductase (NR), glutamine synthetase (GS), and glutamate synthase (GOGAT) were all determined using the detection kits provided by Jiangsu Jingmei Biotechnology Co., Ltd. The detailed measurement method was found in Supplementary Material.

#### 2.3.4. NSC Content and N Content

The stem-sheath samples were collected at heading and maturity from Section 2.3.2. Samples were dried at 105 °C for 30 min, then at 65 °C to constant weight, and ground into powder. Soluble sugar and starch were determined following the method of Wei et al. [20]. NSC content = soluble sugar + starch; NSC accumulation in the stem (mg plant^−1^) = Stem sheath biomass × NSC content; NSC translocation in the stem sheath (mg plant^−1^) = NSC accumulation at heading − NSC accumulation at maturity; NSC translocation rate (%) = (NSC accumulation at heading − NSC accumulation at maturity)/NSC accumulation at heading × 100%. Additionally, the N concentration in each organ at maturity was quantified by the Kjeldahl procedure according to Wei et al. [20]. Dry samples were digested in concentrated H_2_SO_4_ to transform total N into ammonium sulfate, which was then distilled and titrated for quantification.

#### 2.3.5. Determination of Sodium (Na^+^) and Potassium (K^+^) Content

At the post-heat stress and heading stage, leaf and stem-sheath samples were dried and ground into powder. Approximately 0.1 g of dry samples was digested with concentrated HNO_3_, and then diluted with deionized water. The K^+^ and Na^+^ contents were determined via flame photometer (M410, Sherwood Scientific, Cambridge, UK), following the method described by Li et al. [19].

#### 2.3.6. Gene Expression Determination

To quantify transcript-level shifts across stress intensities and durations, representative photosynthetic, ROS-scavenging Na^+^ and K^+^ transport genes identified in our prior RNA-seq [3] dataset were assayed by qRT-PCR, and the key gene information is shown in Table S1. All primers are listed in Supplementary Table S2. The 2^−ΔΔCT^ method, as described by Livak et al. [21], was used to calculate the relative gene expression levels using the mean value of four replicates. The detailed measurement method is found in Supplementary Material.

#### 2.3.7. Panicle Development and Yield Traits

Fifteen main panicles of uniform size were randomly selected, and the length of the first internode, panicle exsertion, and panicle length were measured. Panicle exsertion (%) = (panicle exsertion length/panicle length) × 100%. Yield and its traits were estimated at maturity. Six plants per treatment were harvested to determine panicle number per plant, grain number per panicle, seed setting rate, 1000-grain weight, and yield per plant (adjusted to 14% moisture content).

### 2.4. Data Analysis

Data processing was performed with Excel 2021 and SPSS 26.0 (SPSS Inc., Chicago, IL, USA); statistical evaluation included analysis of variance (ANOVA). For multiple pairwise comparisons among treatments, Tukey’s Honest Significant Difference (HSD) test was performed at the *p* < 0.05 significance level, to strictly control the Type I error rate in multiple comparisons. To test the main effects of salt, heat stress, and their interaction effect on all measured parameters, a two-way ANOVA with a completely randomized 2 × 2 factorial design was performed. A significant salt × heat interaction effect (*p* < 0.05) was considered the statistical basis for the presence of a synergistic effect between salt and heat stress; for variables with a non-significant interaction (*p* > 0.05), the combined effect was described as an additive or more severe combined effect. Principal component analysis (PCA) was performed using the built-in PCA analysis plug-in of Origin 2023 (OriginLab Corp., Northampton, MA, USA). Given that the variables included in the analysis have different scales, units, and numerical ranges, Z-score standardization (mean centering and variance scaling) was strictly performed on all variables via the plug-in’s built-in standard preprocessing function before formal analysis, to eliminate the interference of dimension differences on principal component weighting and analysis results. Graphs were drawn using Origin 2023 (OriginLab Corp., Northampton, MA, USA).

## 3. Results

### 3.1. Effects of Salt and Heat Stress on Oxidative Stress

**Figure 1:**
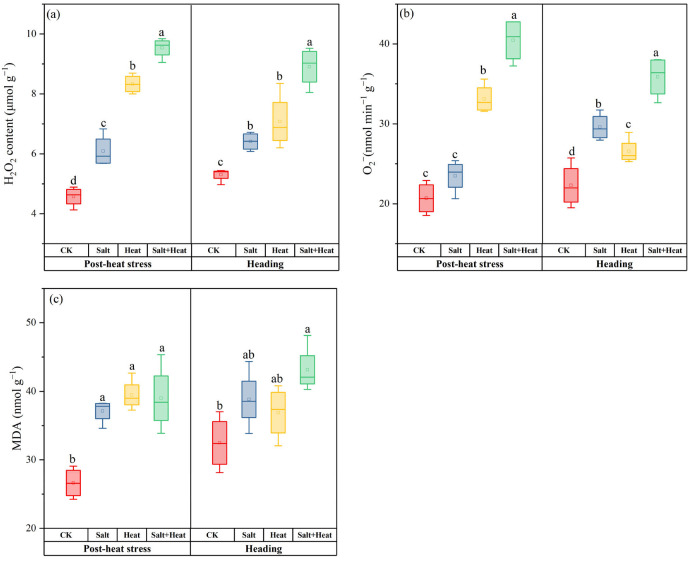
Effect of individual and combined heat and salt stress on (**a**) H_2_O_2_, (**b**) O_2_^−^, and (**c**) MDA contents at post-heat stress and heading stage. Data are mean ± SE (*n* = 4, where *n* represents the number of independent experimental units (biological replicates) per treatment). Different lowercase letters indicate significant differences among treatments at the *p* < 0.05 level by Tukey’s HSD test.

Combined salt + heat stress induced the most severe oxidative stress, with significantly higher ROS accumulation and oxidative damage than single stresses (Figure 1). Post-heat stress, H_2_O_2_ content, and O_2_^−^ production rate in salt + heat stress were 1.3 and 1.5 times higher than CK, and 14.5% and 22.1% higher than heat stress, and 56.6% and 72.2% higher than salt stress. The MDA content in salt + heat stress was 48.3% higher than CK, indicating extensive membrane lipid peroxidation under combined stress conditions. At heading, the combined heat + salt stress was comparatively lower in individual stress than in the combined. Specifically, H_2_O_2_ content and O_2_^−^ generation rate in salt + heat stress were 67.9% and 60.8% higher than CK. Individual heat and salt stress also enhanced the H_2_O_2_ and O_2_^−^ contents more than CK, but their values remained relatively lower than those under the combined salt + heat stress treatment. At the post-heat stress stage, the salt × heat interaction effect was significant for H_2_O_2_ content and O_2_^−^ (*p* < 0.05). At the heading stage, the salt × heat interaction effect was not significant for all oxidative stress-related parameters (*p* > 0.05).

### 3.2. Effects of Salt and Heat Stress on Antioxidant Enzyme Activities

**Figure 2:**
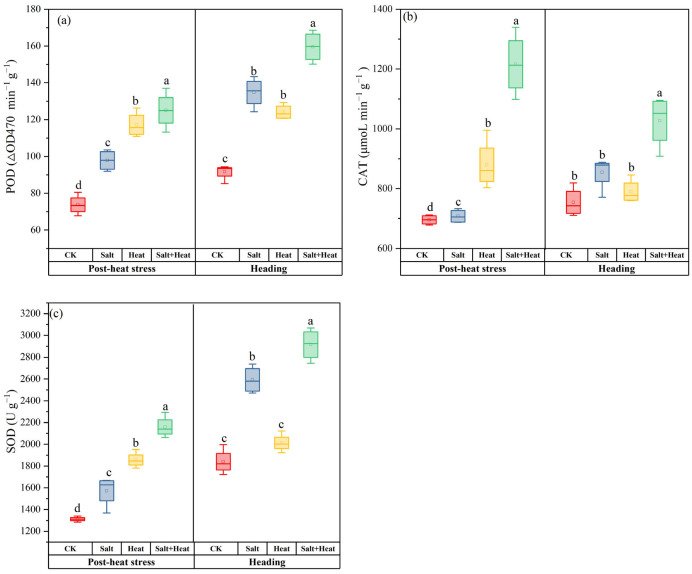
Effect of individual and combined heat and salt stress on (**a**) POD, (**b**) CAT, and (**c**) SOD activities at the post-heat stress and heading stage. Data are mean ± SE (*n* = 4, where *n* represents the number of independent experimental units (biological replicates) per treatment). Different lowercase letters indicate significant differences among treatments at the *p* < 0.05 level by Tukey’s HSD test.

At post-heat stress, the antioxidant enzyme activities in salt + heat stress were markedly increased. For example, the activities of SOD, POD, and CAT were 64.6%, 69.5%, and 74.8% higher than CK, respectively (Figure 2). At heading, the SOD, POD, and CAT were increased by 58.5%, 52.3%, and 36.2%, respectively. Moreover, heat stress showed lower CAT and POD activities than salt stress, but the difference was not significant. At the post-heat stress stage, the salt × heat interaction effect was significant for SOD and POD activity (*p* < 0.05), showing a synergistic induction effect of combined stress on these two antioxidant enzymes; while the salt × heat interaction effect was not significant for CAT activity (*p* > 0.05), with an additive induction effect under combined stress.

### 3.3. Effects of Salt and Heat Stress on Photosynthetic Traits and Aboveground Biomass

**Figure 3:**
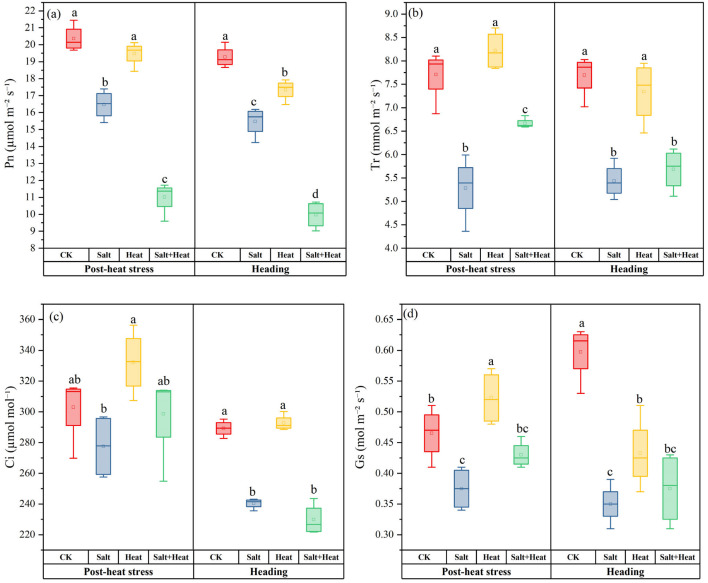
Effect of individual and combined heat and salt stress on (**a**) Pn, (**b**) Tr, (**c**) Ci, and (**d**) Gs at the post-heat stress and heading stage. Data are mean ± SE (*n* = 4, where *n* represents the number of independent experimental units (biological replicates) per treatment). Different lowercase letters indicate significant differences among treatments at the *p* < 0.05 level by Tukey’s HSD test.

The salt + heat stress substantially reduced the net photosynthesis (P_n_) and aboveground dry biomass in rice (Figure 3). At post-heat stress, the P_n_ in salt + heat stress reduced by 45.9% relative to CK. The P_n_ under heat stress treatment remained statistically similar (*p* > 0.05) to CK but was significantly higher than salt stress. At heading, P_n_ was 10.0%, 19.7%, and 48.2% lower under heat, salt, and combined salt + heat stress, as compared with CK. Furthermore, the Tr and C_i_ under heat stress treatment remained statistically similar (*p* > 0.05) to CK but were substantially higher than under salt treatment. For the P_n_, the salt × heat interaction effect was extremely significant at both post-heat stress and heading stages (*p* < 0.01).

Combined salt and heat stress significantly altered the dry matter accumulation pattern and translocation efficiency in rice (Figure S3). At the heading stage, the leaf biomass of rice under combined salt and heat stress was reduced by 16.3% compared with CK, while the stem sheath and aboveground biomass were decreased by 31.3% and 32.1%, respectively. At maturity, no difference was found in leaf biomass among all treatments, whereas the salt + heat stress resulted in reduced stem sheath and aboveground biomass compared to individual stress. In addition, the leaf dry matter and stem-sheath dry matter translocation of rice under combined salt and heat stress were lower than those under individual stress and the CK. The salt × heat interaction effect was extremely significant for aboveground biomass at maturity, stem-sheath biomass at heading and maturity, and stem-sheath biomass translocation (*p* < 0.01), showing a synergistic inhibition effect.

### 3.4. Effects of Salt and Heat Stress on N Accumulation and Translocation

**Figure 4:**
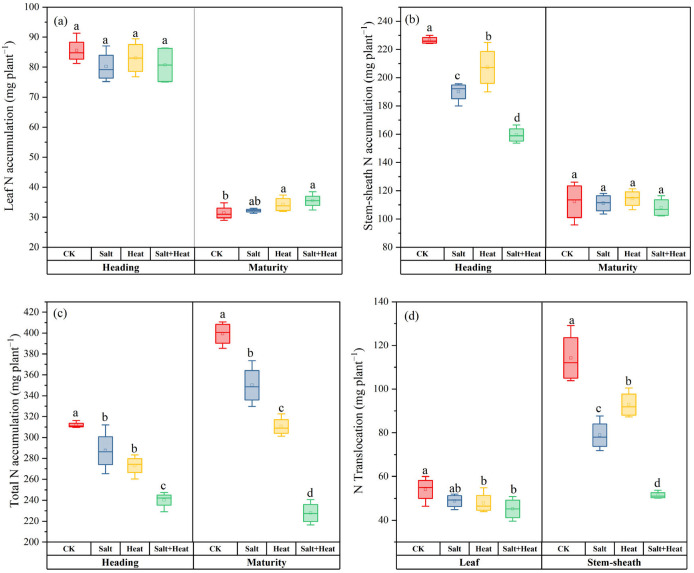
Effect of individual and combined heat and salt stress on (**a**) Leaf N accumulation, (**b**) Stem-sheath accumulation, (**c**) Total N accumulation, and (**d**) N translocation at leaf and stem sheath. Data are mean ± SE (*n* = 4, where *n* represents the number of independent experimental units (biological replicates) per treatment). Different lowercase letters indicate significant differences among treatments at the *p* < 0.05 level by Tukey’s HSD test.

The salt + heat stress treatment significantly altered N accumulation and translocation in rice (Figure 4). Total N accumulation in the whole plant at maturity was substantially reduced by salt + heat stress treatment, and the inhibitory effect was more pronounced under combined stress than under individual stress treatment. For organ-specific total N accumulation per plant, leaf N accumulation at the heading stage showed no significant difference among all treatments, whereas leaf N accumulation under salt + heat stress treatment was increased by 12.9% compared with CK at maturity. In contrast, stem-sheath N accumulation at the heading stage was significantly lower under stress conditions, with salt + heat stress treatment showing a 29.6% reduction compared with CK, whereas salt and heat stress treatments decreased by 16.1% and 8.5%, respectively. However, at maturity, no significant difference was found among all treatments in stem-sheath N accumulation. The total N translocation amount from leaf to grain under salt + heat stress treatment was significantly lower than that of CK. Similarly, the stem-sheath total N translocation in salt + heat stress treatment decreased by 54.9% compared with CK, while single salt and heat stress treatment caused reductions in N translocation of up to 31.0% and 18.7%, respectively. The salt × heat interaction effect was significant for total N accumulation at maturity (*p* < 0.05), indicating a synergistic inhibition of total N accumulation under combined stress.

### 3.5. Effects of Salt and Heat Stress on Nitrogen-Related Metabolism Enzymes

**Figure 5:**
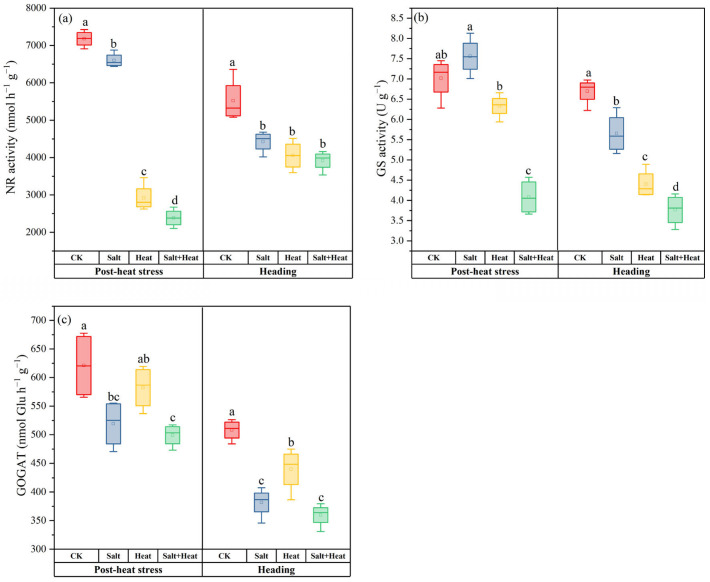
Effect of individual and combined heat and salt stress on nitrogen-related metabolism enzymes, i.e., (**a**) NR, (**b**) GS, and (**c**) GOGAT activity at the post-heat stress and heading stage. Data are mean ± SE (n = 4, where n represents the number of independent experimental units (biological replicates) per treatment). Different lowercase letters indicate significant differences among treatments at the *p* < 0.05 level by Tukey’s HSD test.

The salt + heat stress treatment exerted significant and stage-specific effects on the activities of N assimilation enzymes, i.e., NR, GS, and GOGAT (Figure 5). The NR activity in rice leaves under individual heat and salt stress treatment was reduced by 8.1% and 59.3% compared with the CK at post-heat stress. The combined salt + heat stress led to a more severe reduction of 66.8% in NR activity, whereas at the heading stage, the NR activity in all stress treatments was further decreased relative to CK, with the most pronounced inhibition (29% reduction) being observed in the salt + heat stress treatment.

Furthermore, the GS activity in salt stress treatment was slightly increased by 7.8% relative to CK, while salt + heat stress treatment reduced GS activity by 41.8% at post-heat stress. At the heading stage, the GS activity in CK was the highest, whereas all stress treatments showed a substantial reduction in GS activity. The GOGAT activity in salt + heat stress treatment was reduced by 19.6% compared with CK during post-heat stress. At the heading stage, the GOGAT activity in salt + heat stress treatment was reduced by 29.2%, whereas the reductions in salt and heat stress treatments were 24.9% and 13.5%, as compared to CK. At the post-heat stress stage, the salt × heat interaction effect was extremely significant for GS activity (*p* < 0.01), while it was not significant for NR and GOGAT activities (*p* > 0.05). At the heading stage, the salt × heat interaction effect was significant for NR activity (*p* < 0.05), and was not significant for GS and GOGAT activities (*p* > 0.05)

### 3.6. Effects of Salt and Heat Stress on K^+^ and Na^+^ Content, and K^+^/Na^+^ Ratio in Leaves and Stem Sheaths of Rice

**Figure 6:**
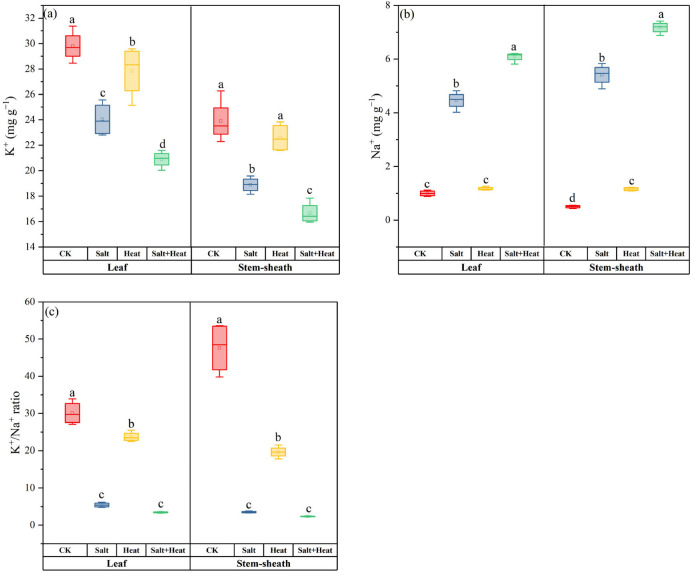
Effect of individual and combined heat and salt stress on (**a**) K^+^ content, (**b**) Na^+^ content, and (**c**) K^+^/Na^+^ ratio, and at the post-heat stress. Data are mean ± SE (*n* = 4, where *n* represents the number of independent experimental units (biological replicates) per treatment). Different lowercase letters indicate significant differences among treatments at the *p* < 0.05 level by Tukey’s HSD test.

At the post-heat stress, salt + heat stress treatment significantly disrupted ion homeostasis in rice, in terms of K^+^, Na^+^ content, and K^+^/Na^+^ ratio between leaves and stem sheaths (Figure 6). For Na^+^ accumulation, both single and combined salt + heat stresses induced a significant increase in Na^+^ content in leaves and stem sheaths compared with the CK. In contrast to Na^+^, K^+^ content in leaves and stem sheaths was significantly reduced by salt and heat stress treatment, with the most severe reduction observed under salt + heat stress treatment. The K^+^/Na^+^ ratio was substantially decreased under stress conditions, with the lowest ratio observed under salt + heat stress treatment. The salt × heat interaction effect was significant for Na^+^ content and K^+^/Na^+^ ratio (*p* < 0.05).

### 3.7. Effects of Salt and Heat Stress on Stem NSC-Related Traits

At the heading stage, both single and combined stresses affected NSC content and accumulation in the stem sheath, but the response patterns differed (Table 1). Relative to CK, single salt stress decreased NSC content by 3.7%, while combined salt + heat stress led to a 4.3% reduction in NSC content. At the heading stage, the NSC accumulation was 9.3%, 10.9%, and 34.3% lower under salt, heat stress, and salt + heat stress treatment than CK. At maturity, stem-sheath NSC content under combined stress treatments was significantly lower than CK, with the most severe reduction observed under salt + heat stress. Furthermore, the NSC translocation rate in CK was 40.24%, which decreased to 33.93%, 35.66%, and 32.75%, respectively, under salt, heat, and salt + heat treatment. The salt × heat interaction effect was extremely significant for NSC accumulation at both heading and maturity stages (*p* < 0.01).

### 3.8. Effects of Salt and Heat Stress on Carbon-Related Metabolic Enzymes

**Figure 7:**
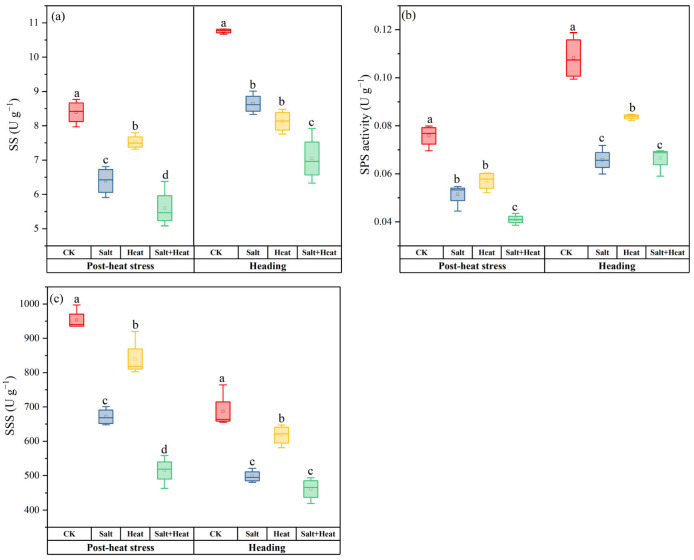
Effect of alt and heat stress on stem carbon-related metabolic enzymes: (**a**) SS, (**b**) SPS, and (**c**) SSS at the post-heat stress and heading stage. Data are mean ± SE (*n* = 4, where *n* represents the number of independent experimental units (biological replicates) per treatment). Different lowercase letters indicate significant differences among treatments at the *p* < 0.05 level by Tukey’s HSD test.

The salt + heat stress treatment induced stage-specific and enzyme-specific responses SPS, SS, and SSS enzyme activities in the stem sheath (Figure 7). Heat stress decreased SPS activity by 10.3% compared with the CK, while salt stress and salt + heat stress caused 23.9% and 33.3% reduction in SPS activity. At the heading stage, the maximum SPS activity was recorded in CK, whereas all stress treatments showed significant reductions in SPS activity. The SS and SSS activity in salt + heat stress was significantly lower than single salt stress, heat stress, and CK during post-heat stress and heading stage. The salt × heat interaction effect was significant for SS and SPS activities at the heading stage (*p* < 0.01).

### 3.9. Effects of Salt and Heat Stress on Relative Gene Expression Levels

**Figure 8:**
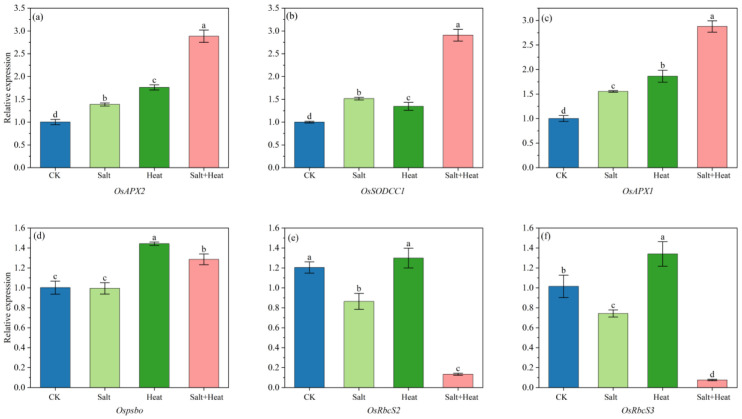
Effect of salt and heat stress on relative expression level of (**a**) *OsAPX2*, (**b**) *OsSODCC1*, (**c**) *OsAPX1*, (**d**) *Ospsbo*, (**e**) *OsRbcS2*, and (**f**) *OsRbcS3* in rice at the post-heat stress using qRT-PCR. Data are mean ± SE (*n* = 4, where *n* represents the number of independent experimental units (biological replicates) per treatment). Different lowercase letters indicate significant differences among treatments at the *p* < 0.05 level by Tukey’s HSD test.

Antioxidant defense-related genes, i.e., *OsAPX2*, *OsSODCC1*, and *OsAPX1*, were significantly upregulated under both single and combined stresses, whereas the upregulation was more pronounced under salt + heat stress treatment (Figure 8). In contrast, photosynthesis-related genes, i.e., *OsRbcS2* and *OsRbcS3*, were predominantly downregulated under salt stress conditions, and the suppression was more severe under combined stress. The expression of *Ospsbo* and *OsRbcS3* was increased under heat stress compared with CK, while no significant difference was found regarding *OsRbcS2* expression between CK and heat stress.

**Figure 9:**
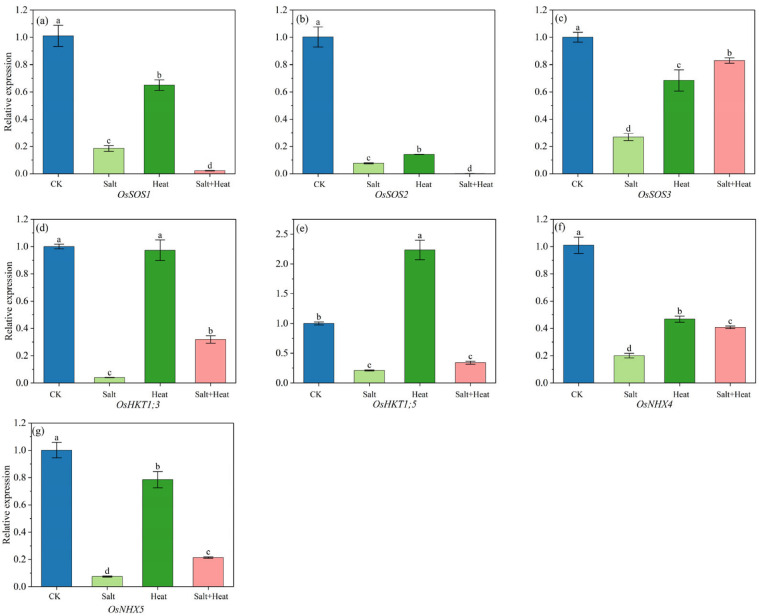
Effect of salt and heat stress on relative expression level of K^+^ and Na^+^ transport genes: (**a**) *OsSOS1*, (**b**) *OsSOS2*, (**c**) *OsSOS3*, (**d**) *OsHKT1;3*, (**e**) *OsHKT1;5*, (**f**) *OsNHX4*, and (**g**) *OsNHX5* in rice at the post-heat stress using qRT-PCR. Data are mean ± SE (*n* = 4, where *n* represents the number of independent experimental units (biological replicates) per treatment). Different lowercase letters indicate significant differences among treatments at the *p* < 0.05 level by Tukey’s HSD test.

Notably, all Na^+^ and K^+^ transport genes, i.e., *OsSOS1*, *OsSOS2*, *OsSOS3*, *OsHKT1;3*, *OsHKT1;5*, *OsNHX4*, and *OsNHX5*, were significantly affected under stress treatments (Figure 9). The SOS pathway genes, i.e., *OsSOS1*, *OsSOS2*, and *OsSOS3*, were significantly downregulated under salt + heat stress, respectively. For the high-affinity K^+^ transporter genes, i.e., *OsHKT1;3* and *OsHKT1;5*, and their relative expressions under salt + heat stress were 68.1% and 65.9% lower than CK. Additionally, the vacuolar Na^+^/H^+^ antiporter genes, i.e., *OsNHX4* and *OsNHX5*, exhibited 59.6% and 78.6% downregulations under salt + heat stress, respectively, which were 2.0- and 2.9-fold higher than those under single salt stress. For antioxidant defense-related genes (*OsAPX2*, *OsSODCC1*, OsAPX1), photosynthesis-related genes (*OsRbcS2*, *OsRbcS3*), and ion transport-related genes (*OsSOS1*, *OsSOS2*, *OsSOS3*, *OsHKT1;3*, *OsHKT1;5*, *OsNHX4*), the salt × heat interaction effect was extremely significant (*p* < 0.01), showing a synergistic regulatory effect of combined stress on the transcription of these genes.

### 3.10. Effects of Salt and Heat Stress on Grain Yield and Its Components in Rice

Grain yield and its components were substantially reduced under salt + heat stress compared with individual stress (Table 2). Compared with CK, salt treatment reduced effective panicles per plant by 14.2%; however, salt + heat stress led to 28.6% reduction, which was substantially lower than that in single salt and heat stress. Similarly, grains per panicle were decreased by 17.6% (salt stress) and 10.0% (heat stress), while salt + heat stress treatment resulted in a 30.3% reduction compared with CK. Compared with CK, 1000-grain weight and seed-setting rate were significantly decreased by salt and heat stress, while salt + heat stress treatment resulted in a severe reduction. Compared to CK, the grain yield was decreased by 34.6% (salt stress) and 20.5% (heat stress), while salt + heat stress led to a 66.4% reduction in grain yield. The salt × heat interaction effect was extremely significant for panicle development traits and final grain yield (*p* < 0.01), significant for 1000-grain weight, seed-setting rate, and productive panicle per plant (*p* < 0.05).

### 3.11. Principal Component Analysis (PCA)

**Figure 10:**
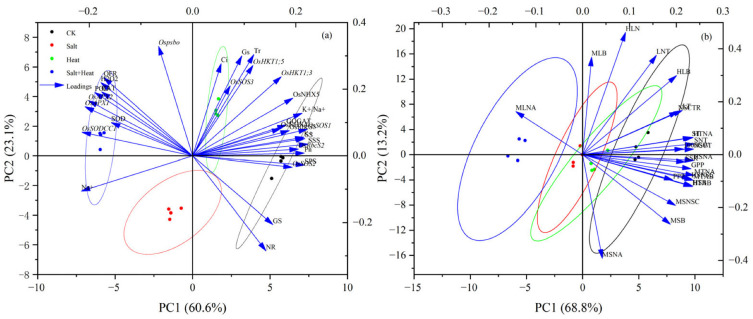
Principal component analysis (PCA) of measured traits: (**a**) PCA of various traits at post-heat stress; (**b**) C and N accumulation and allocation in different plant parts at heading and their translocation to grain.

The first two principal components (PC1 and PC2) explained 60.6% and 23.1% of the total variance, respectively, with a cumulative contribution rate of 83.7% (Figure 10a). The four treatment groups showed a clear separation along the PC1 axis, which represented the core dimension of rice stress response: the CK group was distinctly clustered in the positive direction of PC1, the single salt and single heat stress groups were distributed in the intermediate region, and the combined salt + heat stress group was exclusively clustered in the far negative direction of PC1. This distribution pattern directly confirmed that the combined stress caused a more drastic shift in the overall physiological and molecular state of rice than either single stress, showing a significantly more severe combined effect. The loading plot further revealed two core trait clusters with opposite distribution patterns along the PC1 axis, which corresponded to the key physiological and molecular regulatory pathways revealed in this study. Positive PC1 cluster (traits for normal growth and stress resistance): This cluster was dominated by traits related to ion homeostasis (K^+^/Na^+^ ratio, relative expression of *OsSOS1/2/3*, *OsHKT1;3*, *OsHKT1;5*, *OsNHX4/5*), photosynthetic performance (P_n_, G_s_, Tr, relative expression of *Ospsbo*, *OsRbcS2*, *OsRbcS3*), C-N metabolic activity (activities of NR, GS, GOGAT, SPS, SS, and SSS), and subsequent NSC and nitrogen accumulation and translocation efficiency. Negative PC1 cluster (stress response traits): This cluster was mainly composed of oxidative stress-related traits, including ROS accumulation (H_2_O_2_, O_2_^−^ production rate), membrane lipid peroxidation level (MDA content), antioxidant enzyme activities (SOD, POD, CAT), and relative expression of antioxidant defense-related genes (*OsAPX2*, *OsSODCC1*, *OsAPX1*). These traits were highly positively correlated with each other, and showed a significant negative correlation with the positive PC1 cluster traits.

As illustrated in Figure 10b, the first two principal components (PC1 and PC2) explained 68.8% and 13.2% of the total variance, respectively, with a cumulative contribution rate of 82.0%. Similarly, the four treatment groups were clearly separated along the PC1 axis: the CK group was clustered in the positive direction of PC1, characterized by high NSC and total N accumulation, high translocation efficiency from vegetative organs to panicles, and superior yield component traits; while the combined salt + heat stress group was clustered in the negative direction, with significantly inhibited C-N accumulation and translocation, and severe yield loss.

## 4. Discussion

### 4.1. Combined Salt and Heat Stress Aggravates Oxidative Stress and Photosynthetic Damage

Oxidative homeostasis and photosynthesis are important physiological processes in plants prone to abiotic stresses [22,23,24]. Co-occurrence of two or more stresses may amplify the magnitude of the stress factors, leading to more severe damage in crop plants than a single stress.

In the present study, combined salt + heat stress significantly elevated ROS levels (H_2_O_2_ and O_2_^−^) and disrupted antioxidant enzyme balance, which is in line with the findings of Li et al. [3], who found severe damage in tomato plants under salt and heat stress. It was found that salt + heat stress induced extreme ROS accumulation in rice, whereas the H_2_O_2_ content and O_2_^−^ production rate in salt + heat stress were 1.3 and 1.5 times higher than the CK. This aligns with Rivero et al. [25], who found that combined abiotic stresses often exceed the ROS scavenging capacity of plants, leading to cumulative oxidative damage. Su et al. [26] reported that combined salt + heat stress treatment disrupts the cooperative function of the antioxidant enzyme system, while SOD activity continues to rise, CAT and ascorbate peroxidase (APX) activities were substantially declined, resulting in an imbalance between ROS production and scavenging ability. In the present study, although antioxidant enzymes (SOD, CAT, and POD) were upregulated in salt + heat stress, their activity was insufficient to scavenge excess ROS, resulting in severe membrane damage in terms of lipid peroxidation. Likewise, antioxidant defense-related genes, i.e., *OsAPX2*, *OsSODCC1*, and *OsAPX1*, were upregulated under all stress treatments, but the induction magnitude was most pronounced under salt + heat stress, which indicates that rice plants actively activate the transcriptional expression of antioxidant genes to cope with oxidative stress under combined stress, but the synergistic effect accumulation of ROS under salt + heat stress exceeds the scavenging capacity of the antioxidant system, leading to irreversible cellular damage.

Moreover, ionic imbalance acts as a key mediator through which combined stress amplifies oxidative stress and photosynthetic damage. Na^+^ toxicity directly disrupts chloroplast membrane integrity, while K^+^ deficiency impairs the structural stability of antioxidant enzymes, leading to insufficient ROS scavenging efficiency [27]. Our results showed that combined salt + heat stress treatment significantly decreased leaf K^+^ content, increased Na^+^ content, and consequently reduced the K^+^/Na^+^ ratio. Na^+^ and K^+^ homeostasis was further confirmed by key ion transport genes involved in Na^+^ exclusion, i.e., *OsSOS1*, *OsSOS2*, and *OsSOS3*; Na^+^ compartmentalization genes, i.e., *OsNHX4* and *OsNHX5*; and K^+^ uptake related genes, i.e., *OsHKT1;3* and *OsHKT1;5*, which were significantly downregulated under salt + heat stress. The downregulation of these genes reduced the ability of rice to maintain ion homeostasis, leading to excessive Na^+^ accumulation and K^+^ loss. Alzahrani et al. [28] also found that combined stress causes more oxidative damage than either salt or drought stress alone, primarily because salt-induced Na^+^ overaccumulation and suppressed K^+^ uptake disrupt ion homeostasis, triggering a ROS overproduction and reducing the membrane stability index.

Photosynthetic damage under combined stress is driven by both stomatal and non-stomatal limitations. Salt stress alone induces stomatal closure to reduce ion uptake [5], while heat stress primarily damages the photosynthetic apparatus [16]. High salt (≥100 mM NaCl) combined with heat significantly reduces g_s_ and P_n_, with non-stomatal damage as the dominant factor in tomato [3]. Our results showed similar trends, i.e., salt + heat stress reduced P_n_ by 45.9% and 48.2% during the post-heat and heading stage, respectively, compared with CK. Studies have shown that high temperature directly disrupts the structural integrity of photosystem II, shortens the crop growth cycle, and ultimately hinders plant growth and development [29,30]. Based on previous research on combined salt and heat stress [3], we hypothesize that the imbalance of the oxidative phosphorylation pathway may be a key mechanism underlying the aggravated photosynthetic damage and oxidative stress observed in this study, which may lead to insufficient ATP synthesis, thereby suppressing both ATP-dependent photosynthetic enzyme activity and the energy supply to the ROS-scavenging system. Combined salt + heat stress inhibited photosynthesis in quinoa more than either single stress, mainly by reducing total chlorophyll content and suppressing stomatal conductance. Heat stress aggravates salt-induced ionic imbalance, jointly triggering oxidative stress that disrupts photosynthetic pigment stability and stomatal regulation [31,32]. We found that leaf Tr and G_s_ increased under heat stress treatment, whereas salt stress inhibited Tr, G_s_, and P_n_, indicating fundamental differences in how salt and heat affect photosynthesis. Nevertheless, the photosynthetic inhibition pattern of combined salt + heat stress treatment in alfalfa resembles the effects of salt stress alone [26]. At the molecular level, the photosynthesis-related genes showed divergent responses to different stresses. Specifically, *Ospsbo* and *OsRbcS3* were significantly upregulated under heat stress compared to CK. In contrast, under salt-containing treatments (salt and salt + heat stress), these photosynthetic genes were significantly downregulated, and the suppression was most severe under salt + heat stress. Thus, salt stress may play a dominant role in regulating the photosynthetic physiological processes in rice in response to combined salt + heat stress treatment. It should be noted that chlorophyll fluorescence parameters were not measured in this study, as our work focused on the photosynthetic dark reaction and its downstream carbon–nitrogen metabolic effects. This indicator will be a core focus of our follow-up research to further clarify the photosynthetic damage mechanism of combined salt and heat stress.

### 4.2. Temporal Dynamics of NSCs: Persistent Pre-Anthesis Synthesis Deficit and Further Failure of Post-Anthesis Remobilization

NSCs stored in rice stem sheath are the core carbon reserve for panicle development and grain filling, whose synthesis, accumulation, and remobilization present strict stage-specific temporal dynamics in the rice growth cycle [9,12,33]. After combined salt and heat stress, the first wave of damage to NSC metabolism occurred, which laid the irreversible foundation for the subsequent continuous NSC deficit (Figure 7). The P_n_ under salt + heat stress was significantly reduced, which directly and persistently inhibited the de novo synthesis of sucrose and starch, the core components of NSCs. At the molecular level, combined stress significantly downregulated the expression of photosynthesis-related genes, i.e., *OsPsbO*, *OsRbcS2*, and *OsRbcS3* [3]. Rubisco is the rate-limiting enzyme for plant CO_2_ fixation, and the reduced expression of its small-subunit genes (*OsRbcS2*, *OsRbcS3*) directly inhibits Rubisco activity, leading to insufficient biosynthesis of photosynthates and decreased carbohydrate accumulation in the stem sheath [34,35]. Meanwhile, the synergistic oxidative burst (Figure 1 and Table S3) induced by combined stress further inhibited the activity of key NSC metabolism enzymes: salt stress-induced ROS accumulation has been widely shown to SS and SSS in rice, while heat stress further suppresses these enzymes [36,37]. Our results confirmed that salt + heat stress significantly reduced the activities of SS, SPS, and SSS in the stem sheath, compared with CK after combined salt and heat stress. This persistent inhibition of photosynthetic carbon fixation and key metabolic enzymes directly impaired the basic synthesis capacity of NSCs from the early panicle development stage, which is consistent with previous findings that abiotic stress at panicle initiation reduces pre-anthesis carbohydrate accumulation in rice [9,20].

The present study showed that the combined salt + heat stress had the lowest stem-sheath NSC content and total NSC accumulation at the heading stage, which were significantly lower than CK, respectively, with no transient accumulation observed (Table 1). This significant NSC reserve deficit at the heading stage is the direct phenotypic result of the persistent damage of combined stress to photosynthetic carbon fixation and C metabolism enzymes from panicle initiation to the heading stage. The results were consistent with Wei et al. [20], who found that combined salt and drought stress reduces photosynthetic assimilation and inhibits NSC accumulation in the rice stem sheath. Zhen et al. [9] confirmed that heat-induced photosynthetic damage reduces NSC synthesis in rice.

Our results showed that the NSC translocation amount and translocation rate from the stem sheath to grain under combined stress were significantly lower than CK, respectively, and were also significantly lower than the single salt and heat stress (Table 1). This indicates that the core damage of combined stress to C metabolism is dual, which impairs the NSC “synthesis-remobilization” balance: on the one hand, it causes a significant reduction in pre-anthesis NSC reserve accumulation; on the other hand, it further inhibits the post-anthesis remobilization and translocation of the already limited NSC reserve, which forms a double bottleneck for grain carbon supply.

### 4.3. Combined Stress Triggers Aberrant Whole-Plant Nitrogen Partitioning Dynamics: From Pre-Anthesis Assimilation and Allocation Imbalance to Post-Anthesis Remobilization Blockage

In this study, the impact of combined salt and heat stress on rice N metabolism led to the systematic reshaping of the whole-plant N partitioning pattern, which was underpinned by reduced total N uptake, and severe blockage of post-anthesis N remobilization from vegetative source organs to reproductive sink organs [26,27]. At the panicle initiation stage, the initial inhibition of N uptake and assimilation established the baseline for subsequent partitioning imbalance. Combined stress suppressed root N acquisition: salt-induced Na^+^ overaccumulation competed with inorganic N (NO_3_^−^, NH_4_^+^) uptake via root transporters [38], while heat stress reduced root vitality [39], and the concurrent downregulation of ion homeostasis genes (*OsSOS1/2/3*, *OsHKT1;3*, *OsHKT1;5*, *OsNHX4/5*) further weakened mineral nutrient absorption [27,28]. Meanwhile, the activities of key N assimilation enzymes (NR, GS, GOGAT) in leaves were significantly reduced under combined stress, limiting the conversion of inorganic N into metabolically active organic N [40]. These effects ultimately resulted in a 42.9% reduction in total whole-plant N accumulation at maturity, defining the limited size of the total plant N pool.

Notably, N was not uniformly reduced across vegetative organs even at this stage, but showed a clear survival-prioritized redistribution pattern. At the heading stage, stem-sheath total N accumulation was significantly reduced under combined stress compared with CK, while leaf total N accumulation showed no significant difference among treatments. This organ-specific divergence indicated that under the limited total N pool, rice preferentially allocated available N to leaf tissues (core photosynthetic organs) to maintain basic carbon assimilation, at the cost of N reserve accumulation in stem sheaths (the primary temporary N storage pool for panicle development) [17,41]. This pre-anthesis allocation imbalance directly reduced the N reserve pool available for post-anthesis remobilization.

The most prominent aberrant N redistribution occurred during the grain filling stage (heading to maturity), characterized by a near-complete blockage of N remobilization from vegetative organs to grains. In rice, the majority of grain nitrogen (N) comes from the remobilization of pre-stored N in vegetative organs after heading [42], but our results showed that combined stress reduced stem-sheath-to-grain N translocation by 54.9% compared with CK, with a similarly significant reduction in leaf-to-grain N translocation. This remobilization blockage drove two key redistribution phenotypes: (1) N that should have been transported to grains was trapped in senescing leaves, resulting in a 12.9% increase in total leaf N accumulation at maturity under combined stress; (2) stem-sheath N accumulation, which was significantly lower at heading, showed no significant difference from CK at maturity, confirming the remobilization failure of pre-stored N.

### 4.4. C and N Deficiency Inhibits Panicle Development and Reduces Yield

Our results showed that combined salt and heat stress significantly reduced the N remobilization rate in rice, causing severe blockage of N translocation from vegetative organs to grains. This blockage not only led to increased total N retention in mature leaves and stem sheaths but also persistently reduced the availability of metabolically active, remobilizable N in leaves from the heading stage onward (Figure 4 and Figure 5). The reduced leaf N availability further disrupted C-N metabolic balance, inhibited photosynthetic carbon assimilation, and ultimately decreased NSC accumulation and effective substrate supply to developing grains (Table 2 and Figure 4).

These findings are consistent with the established source-sink theory that reproductive sink activity is the primary determinant of rice yield under combined abiotic stress [43], as grain filling depends on both post-anthesis photosynthate allocation and pre-stored C/N remobilization from vegetative organs [9,18]. In this study, the severe limitation of both source-end C/N substrate supply and translocation to the panicle sink under combined salt and heat stress directly explains the inhibited grain filling and yield reduction in rice. The blocked transport of NSCs and N is attributed to the synergistic adverse effects of salt and heat on panicle development. Salt + heat stress reduced grains per panicle and seed-setting rate, leading to reduced sink intensity (Table S3). Under combined stress, the delayed panicle exsertion and sharp reduction in spikelet number transformed vegetative organs into temporary “alternative sinks” that stored excess NSCs and N, further inhibiting the transport of assimilates to reproductive organs [44,45]. Concurrently, the decreased sink capacity conversely suppressed leaf photosynthesis, forming a vicious cycle between source and sink limitation.

Moreover, beyond sink constraints, the activity of C and N metabolism enzymes and the expression of key regulatory genes are involved in mediating NSC and N transport [46,47]. The SS and SSS are key enzymes for converting sucrose to starch in developing panicles [48], and their persistent low activity under salt + heat stress inhibited starch synthesis in spikelets, which was correlated with reduced NSC accumulation in panicles. Similarly, N metabolism enzymes (NR, GS, and GOGAT) are critical for synthesizing amino acids (e.g., glutamine) that regulate panicle filling [41], and their failed recovery after stress reduced the transport efficiency of N and further disrupted the C-N balance. Together, this “substrate-energy double bottleneck” inhibited panicle development at multiple levels, i.e., from spikelet differentiation to grain filling, ultimately leading to a significant yield loss under combined salt and heat stress.

Notably, this study only used one widely cultivated commercial rice variety as the research material, which has certain limitations. Existing studies have confirmed that rice genotypes with different stress tolerances show significantly different physiological, metabolic, and transcriptional responses to combined stress, and the yield penalty caused by combined stress also varies greatly among different genotypes [18,27,49]. For instance, salt- and heat-tolerant rice genotypes can maintain higher antioxidant enzyme activity, photosynthetic efficiency, and C-N metabolic homeostasis under combined stress, thus showing lower spikelet sterility and yield loss than sensitive genotypes [18,38]. In follow-up studies, we will screen and compare contrasting rice genotypes with significant differences in tolerance to combined salt and heat stress, to further verify the regulatory mechanism revealed in this study, and mine the key stress-responsive genes and core metabolic pathways that determine the differential tolerance of rice to combined salt and heat stress. This will not only deepen the understanding of the genetic basis of rice multi-stress tolerance, but also provide more precise molecular targets and elite germplasm resources for breeding rice varieties with high and stable yield in saline–alkali land under global warming.

## 5. Conclusions

Combined salt and heat stress exerts a more severe combined effect than individual salt or heat stress alone. Specifically, combined stress intensified oxidative stress, disrupted Na^+^/K^+^ ion homeostasis, and caused persistent impairment of the photosynthetic apparatus in rice, and these physiological damages are tightly driven by the coordinated misregulation of antioxidant defense, ion transport, and photosynthesis-related key genes identified in this study. These molecular perturbations suppressed carbon and nitrogen metabolism, decreased NSC accumulation in stem-sheath tissues, and resulted in greater yield loss than individual stresses. More precisely, this molecular–physiological cascade induced dual damage to carbon metabolism (persistent pre-anthesis NSC synthesis deficit and post-anthesis remobilization failure) and coupled nitrogen redistribution disorder (inhibited post-anthesis nitrogen translocation to developing grains), which severely disrupted the source-sink coupling of carbon and nitrogen nutrition, and ultimately drove the substantial synergistic yield loss under combined stress. Collectively, this study clarifies the core regulatory role of carbon and nitrogen metabolic dynamics in rice response to combined salt and heat stress and provides a solid theoretical basis for stress-tolerant rice breeding and targeted cultivation management in saline areas.

## Figures and Tables

**Table 1:** Effects of salt and heat stress on stem NSC-related traits.

Treatments	NSC Content at Heading (mg g^−1^)	NSC Content at Maturity (mg g^−1^)	NSC Accumulation at Heading (g Plant^−1^)	NSC Accumulation at Maturity (g Plant^−1^)	NSC Translocation (g Plant^−1^)	NSC Translocation Rate (%)
CK	204.98 ± 3.94 ^b^	163.91 ± 2.98 ^a^	6.96 ± 0.36 ^a^	4.16 ± 0.27 ^a^	2.80 ± 0.23 ^a^	40.24 ± 2.51 ^a^
Salt	197.26 ± 2.49 ^c^	156.06 ± 3.37 ^b^	6.31 ± 0.17 ^b^	4.17 ± 0.19 ^a^	2.14 ± 0.11 ^b^	33.93 ± 1.79 ^b^
Heat	216.37 ± 3.10 ^a^	163.97 ± 3.73 ^a^	6.20 ± 0.13 ^b^	3.99 ± 0.10 ^a^	2.21 ± 0.13 ^b^	35.66 ± 1.66 ^b^
Salt + Heat	196.10 ± 3.39 ^c^	148.46 ± 6.00 ^c^	4.57 ± 0.16 ^c^	3.07 ± 0.03 ^b^	1.50 ± 0.16 ^c^	32.75 ± 1.07 ^c^

Note: Different lowercase letters indicate significant differences among treatments at the *p* < 0.05 level by Tukey’s HSD test.

**Table 2:** Effects of salt and heat stress on rice yield and its components.

Treatments	Panicle Exsertion (%)	Productive Panicle per Plant	Grains per Panicle	Seed-Setting Rate (%)	1000-Grain Weight (g)	Grain Yield (g Plant^−1^)
CK	96.8 ± 1.4 ^a^	12.3 ± 0.9 ^a^	128.6 ± 8.2 ^a^	81.7 ± 4.3 ^a^	21.1 ± 0.8 ^a^	27.1 ± 1.6 ^a^
Salt stress	70.4 ± 4.5 ^c^	10.5 ± 0.6 ^b^	106.0 ± 2.6 ^c^	75.8 ± 1.6 ^b^	18.2 ± 0.5 ^b^	17.7 ± 1.0 ^c^
Heat stress	82.5 ± 5.5 ^b^	12.0 ± 0.8 ^a^	115.2 ± 2.6 ^b^	75.9 ± 4.3 ^b^	17.6 ± 0.6 ^b^	21.6 ± 3.0 ^b^
Salt + heat stress	55.4 ± 10.4 ^d^	8.8 ± 0.9 ^c^	89.4 ± 7.1 ^d^	59.7 ± 3.4 ^c^	16.0 ± 0.6 ^c^	9.1 ± 0.8 ^d^

Note: Data are mean ± SE (*n* = 4, where *n* represents the number of independent experimental units (biological replicates) per treatment). Different lowercase letters indicate significant differences among treatments at the *p* < 0.05 level by Tukey’s HSD test.

## Data Availability

The original contributions presented in this study are included in the article/Supplementary Material. Further inquiries can be directed to the corresponding authors.

## Supplementary Materials

The following supporting information can be downloaded at: https://www.mdpi.com/article/10.3390/antiox15030308/s1, Table S1. Information on key genes in photosynthesis, ROS, Na^+^, and K^+^ in response; Table S2. qRT-PCR primer sequence information; Table S3. Significance of rice physiological parameters under salt and heat stress; Figure S1. Photos of rice at the heading stage; Figure S2. The maximum and minimum temperature and average daily solar radiation during the experiment; Figure S3. Aboveground biomass and translocation at the heading and maturity stage. (a) Leaf biomass; (b) Stem sheath biomass; (c) Aboveground biomass; (d) Aboveground biomass translocation.
