# Supplementary material for: Combined Salt and Heat Stress Aggravates Oxidative Stress and Photosynthetic Damage, Disrupting Carbon and Nitrogen Metabolism and Yield in Rice
Source: Antioxidants (Basel). 2026 Feb 28;15(3):308. doi: 10.3390/antiox15030308 (PMC13023559; doi:10.3390/antiox15030308)
Supplement: Supplementary file 1 [file antioxidants-15-00308-s001.zip › antioxidants-4141228-supplementary.pdf]

Supplementary Material S1

2.3.1 Determination of Antioxidant Enzyme Activities

2.3.1.1 Peroxidase (POD) Activity Assay

POD activity was determined using a commercial kit (Catalog No. ADS-W-KY003-48).

Assay principle: POD catalyzes the oxidation of guaiacol by H_2O_2 to produce a reddish-brown product, which has a maximum absorption peak at 470 nm. POD activity was quantified by measuring the change in absorbance at 470 nm per unit time.

Assay procedure: The 200 μL reaction system contained 10 μL of sample supernatant, 40 μL Reagent 1, 140 μL Reagent 2, and 10 μL Reagent 3. After mixing, the absorbance at 470 nm was read immediately (A1) and again after 1 min (A2) using a microplate reader, with ΔA = A2 - A1.

Activity unit definition: One unit (U) of POD activity was defined as an increase of 1 in absorbance at 470 nm per minute per gram of fresh tissue (or per milligram of protein) in the reaction system.

Normalization method: POD activity was normalized to sample fresh weight (expressed as ΔOD_{470}/min/g fresh weight) and total protein concentration (expressed as ΔOD_{470}/min/mg protein), respectively. All assays were performed with four independent biological replicates per treatment.

2.3.1.2 Superoxide Dismutase (SOD) Activity Assay

SOD activity was determined using a WST-8 method commercial kit (Catalog No. ADS-W-KY011-48).

Assay principle: WST-8 reacts with superoxide anions ($O_2{\cdot}^-$) generated by the xanthine oxidase coupling system to produce a water-soluble formazan dye with a maximum absorption peak at 450 nm. SOD scavenges $O_2{\cdot}^-$ and thus inhibits the formation of formazan; the darker the reaction solution, the lower the SOD activity, and vice versa.

Assay procedure: The 200 μL reaction system was set up according to the kit instructions, including sample tube, sample control tube, blank control 1, and blank control 2. After mixing all reagents, the reaction mixture was incubated in the dark at 25°C for 30 min, and the absorbance at 450 nm was measured using a microplate reader. The inhibition rate of SOD on the $O_2{\cdot}^-$ reaction was calculated for each sample.

Activity unit definition: One unit (U) of SOD activity was defined as the amount of enzyme required to achieve 50% inhibition of the $O_2{\cdot}^-$ coupling reaction in the reaction system.

Normalization method: SOD activity was normalized to sample fresh weight (expressed as U/g fresh weight) and total protein concentration (expressed as U/mg protein), respectively. All assays were performed with four independent biological replicates per treatment.

2.3.1.3 Catalase (CAT) Activity Assay

CAT activity was determined using a commercial kit (Catalog No. ADS-W-KY002-48).

Assay principle: CAT catalyzes the decomposition of H_2O_2 into H_2O and O_2. The residual H_2O_2 reacts with a specific chromogenic probe to form a colored product with a maximum absorption peak at 510 nm. CAT activity was calculated based on the reduction of H_2O_2 in the reaction system.

Assay procedure: The reaction was initiated by mixing 10 μL of sample supernatant with 70 μL Reagent 1 and 20 μL Reagent 2, and incubated accurately at 25°C for 5 min. The reaction was terminated by adding 100 μL Reagent 3, then 10 μL of the mixture was used for the chromogenic reaction. The absorbance at 510 nm was measured using a microplate reader, with ΔA = A_blank - A_sample.

Activity unit definition: One unit (U) of CAT activity was defined as the amount of enzyme that catalyzes the decomposition of 1 μmol of H_2O_2 per minute per gram of fresh tissue (or per milligram of protein) at 25°C.
Normalization method: CAT activity was normalized to sample fresh weight (expressed as μmol/min/g fresh weight) and total protein concentration (expressed as μmol/min/mg protein), respectively. All assays were performed with four independent biological replicates per treatment.

2.3.3. C and N Metabolism Enzyme Activity

2.3.3.1 Nitrate Reductase (NR) Activity Assay

NR activity was determined using an in vitro method commercial kit (Catalog No. ADS-W-N001).
Assay principle: NR catalyzes the reduction of nitrate to nitrite. The generated nitrite reacts quantitatively with sulfanilamide and α-naphthylamine to form a purplish-red azo compound with a maximum absorption peak at 530 nm. NR activity was quantified by measuring the absorbance at 530 nm.
Assay procedure: The reaction system included 20 μL of sample supernatant, 70 μL Reagent 1, and 30 μL Reagent 2 for the assay tube, with distilled water instead of Reagent 1 and Reagent 2 for the control tube. The mixture was incubated in the dark at 30°C for 30 min, then 100 μL of chromogenic mixture (Reagent 3 + Reagent 4, 1:1 v/v) was added. After incubation at 30°C for 15 min in the dark, the absorbance at 530 nm was measured, with $\Delta A = A\_assay - A\_control$.
Activity unit definition: One unit of NR activity was defined as the amount of enzyme that catalyzes the production of 1 nmol of nitrite per hour per gram of fresh tissue (or per milligram of protein).
Normalization method: NR activity was normalized to sample fresh weight (expressed as nmol/h/g fresh weight) and total protein concentration (expressed as nmol/h/mg protein), respectively. All assays were performed with four independent biological replicates per treatment.

2.3.3.2 Glutamine Synthetase (GS) Activity Assay

GS activity was determined using a commercial kit (Catalog No. ADS-W-N008).
Assay principle: In the presence of ATP and Mg^{2+}, GS catalyzes the synthesis of glutamine from ammonium ions and glutamate. The product is further converted to γ-glutamyl hydroxamic acid, which forms a colored complex in acidic conditions with a maximum absorption peak at 540 nm. GS activity was quantified by measuring the absorbance at 540 nm.
Assay procedure: The reaction system included 200 μL of sample supernatant, 320 μL Reagent 2, and 120 μL Reagent 3 for the assay tube, with Reagent 1 instead of Reagent 2 for the control tube. After mixing, the mixture was incubated in a 37°C water bath for 30 min, then 200 μL Reagent 4 was added to terminate the reaction. After centrifugation at 8000 rpm for 10 min at 4°C, the absorbance of the supernatant at 540 nm was measured, with $\Delta A = A\_assay - A\_control$.
Activity unit definition: One unit (U) of GS activity was defined as the amount of enzyme that causes a change of 0.005 in absorbance at 540 nm per minute per gram of fresh tissue (or per milligram of protein).
Normalization method: GS activity was normalized to sample fresh weight (expressed as U/g fresh weight) and total protein concentration (expressed as U/mg protein), respectively. All assays were performed with four independent biological replicates per treatment.

2.3.3.3 Glutamate Synthase (GOGAT) Activity Assay

Fd-GOGAT activity was determined using a commercial kit (Catalog No. ADS-W-N002-48).
Assay principle: Fd-GOGAT catalyzes the transfer of the amide group from glutamine to

α-ketoglutarate to form two molecules of glutamate. The generated glutamate is decomposed by a specific enzyme complex and reacts with a chromogenic reagent to form a yellow product with a maximum absorption peak at 450 nm. Fd-GOGAT activity was quantified by measuring the absorbance at 450 nm.

Assay procedure: The enzymatic reaction system included 100 μL of sample supernatant, 50 μL Reagent 1, 50 μL Reagent 2, 50 μL Reagent 3, and 50 μL Reagent 4 mix for the assay tube, with distilled water instead of Reagent 2 for the control tube. After incubation at 30°C for 30 min, the reaction was terminated by boiling in a 95°C water bath for 5 min. After centrifugation, the supernatant was used for the chromogenic reaction, and the absorbance at 450 nm was measured, with $\Delta A = A\_assay - A\_control$.

Activity unit definition: One unit of Fd-GOGAT activity was defined as the amount of enzyme that catalyzes the production of 1 nmol of glutamate per hour per gram of fresh tissue (or per milligram of protein).

Normalization method: Fd-GOGAT activity was normalized to sample fresh weight (expressed as nmol Glu/h/g fresh weight) and total protein concentration (expressed as nmol Glu/h/mg protein), respectively. All assays were performed with four independent biological replicates per treatment.

2.3.3.4 Sucrose Phosphate Synthase (SPS) Activity Assay

SPS activity was determined using a commercial plant SPS ELISA kit (Catalog No. S930970-50T/EA).

Assay principle: A double-antibody one-step sandwich ELISA method was applied. Microplates were pre-coated with a monoclonal antibody specific for plant SPS. Sample supernatant, SPS standard solutions, and horseradish peroxidase (HRP)-labeled detection antibody were sequentially added to the microwells. After incubation and thorough washing, the chromogenic substrate tetramethylbenzidine (TMB) was added for color development. TMB is converted to a blue product under HRP catalysis, and further to a stable yellow product under acidic conditions. The absorbance (OD value) at 450 nm is positively correlated with SPS activity in the sample, which was used to calculate the enzyme activity of each sample via a standard curve.

Assay procedure: The assay was performed strictly following the manufacturer’s instructions. Briefly, 10 μL of sample supernatant (5-fold diluted with sample diluent) or 50 μL of SPS standard solutions (0, 0.5, 1, 2, 4, 8 U/L) were added to the corresponding wells, followed by 100 μL of HRP-labeled detection antibody. The plates were sealed with adhesive films and incubated at 37°C for 60 min. After 5 rounds of washing with wash buffer, 50 μL of chromogen solution A and 50 μL of chromogen solution B were added to each well, followed by incubation at 37°C in the dark for 15 min. The reaction was terminated by adding 50 μL of stop solution, and the absorbance at 450 nm was measured within 15 min using a microplate reader. A standard curve was constructed with standard concentration as the abscissa and corresponding OD value as the ordinate, and the SPS activity of each sample was calculated via the linear regression equation of the standard curve.

Activity unit definition: The enzyme activity unit was defined as U/L, with a linear detection range of 0.1–8 U/L and a minimum detection limit of 0.1 U/L.

Normalization method: SPS activity was normalized to sample fresh weight (expressed as U/g fresh weight) and total protein concentration (expressed as U/mg protein), respectively. The final activity was corrected for the 5-fold sample dilution factor during the assay. All assays were performed with four independent biological replicates per treatment.

2.3.3.5 Sucrose Synthase (SuS) Activity Assay

SuS activity was determined using a commercial plant SuS ELISA kit (Catalog No. S930404-100T/EA).

Assay principle: A double-antibody one-step sandwich ELISA method was applied. Microplates were pre-coated with a monoclonal antibody specific for plant SuS. Sample supernatant, SuS standard solutions, and HRP-labeled detection antibody were sequentially added to the microwells. After incubation and thorough washing, TMB was added for color development. The absorbance at 450 nm is positively correlated with SuS activity in the sample, which was used to calculate the enzyme activity of each sample via a standard curve.

Assay procedure: The assay was performed strictly following the manufacturer's instructions. Briefly, 10 μL of sample supernatant (5-fold diluted with sample diluent) or 50 μL of SuS standard solutions (0, 6, 12, 24, 48, 96 U/mL) were added to the corresponding wells, followed by 100 μL of HRP-labeled detection antibody. The plates were sealed and incubated at 37°C for 60 min. After 5 rounds of washing, 50 μL of chromogen solution A and 50 μL of chromogen solution B were added to each well, followed by incubation at 37°C in the dark for 15 min. The reaction was terminated by adding 50 μL of stop solution, and the absorbance at 450 nm was measured within 15 min using a microplate reader. A standard curve was constructed with standard concentration as the abscissa and corresponding OD value as the ordinate, and the SuS activity of each sample was calculated via the linear regression equation of the standard curve.

Activity unit definition: The enzyme activity unit was defined as U/mL, with a linear detection range of 1–96 U/mL and a minimum detection limit of 0.1 U/mL.

Normalization method: SuS activity was normalized to sample fresh weight (expressed as U/g fresh weight) and total protein concentration (expressed as U/mg protein), respectively. The final activity was corrected for the 5-fold sample dilution factor during the assay. All assays were performed with four independent biological replicates per treatment.

2.3.3.6 Soluble Starch Synthase (SSS) Activity Assay

SSS activity was determined using a commercial plant SSS ELISA kit (Catalog No. S930607-100T/EA).

Assay principle: A double-antibody one-step sandwich ELISA method was applied. Microplates were pre-coated with a monoclonal antibody specific for plant SSS. Sample supernatant, SSS standard solutions, and HRP-labeled detection antibody were sequentially added to the microwells. After incubation and thorough washing, TMB was added for color development. The absorbance at 450 nm is positively correlated with SSS activity in the sample, which was used to calculate the enzyme activity of each sample via a standard curve.

Assay procedure: The assay was performed strictly following the manufacturer's instructions. Briefly, 10 μL of sample supernatant (5-fold diluted with sample diluent) or 50 μL of SSS standard solutions (0, 50, 100, 200, 400, 800 U/L) were added to the corresponding wells, followed by 100 μL of HRP-labeled detection antibody. The plates were sealed and incubated at 37°C for 60 min. After 5 rounds of washing, 50 μL of chromogen solution A and 50 μL of chromogen solution B were added to each well, followed by incubation at 37°C in the dark for 15 min. The reaction was terminated by adding 50 μL of stop solution, and the absorbance at 450 nm was measured within 15 min using a microplate reader. A standard curve was constructed with standard concentration as the abscissa and corresponding OD value as the ordinate, and the SSS activity of each sample was

calculated via the linear regression equation of the standard curve.

Activity unit definition: The enzyme activity unit was defined as U/L, with a linear detection range of 1.0–800 U/L and a minimum detection limit of 1.0 U/L.

Normalization method: SSS activity was normalized to sample fresh weight (expressed as U/g fresh weight) and total protein concentration (expressed as U/mg protein), respectively. The final activity was corrected for the 5-fold sample dilution factor during the assay. All assays were performed with four independent biological replicates per treatment.

2.3.6 Gene Expression Quantification via Quantitative Real-Time PCR (qRT-PCR)

2.3.6.1 Total RNA Extraction and Quality Control

Total RNA was extracted from frozen top fully expanded rice leaves (sampled immediately after heat stress treatment) using a column-based plant total RNA extraction kit, following the manufacturer's optimized protocol. Briefly, 0.1 g of tissue was ground to fine powder in liquid nitrogen, lysed, filtered, and mixed with anhydrous ethanol, then transferred to a purification column for sequential washing and final elution with RNase-free water. RNA integrity was verified via 1.2% agarose gel electrophoresis (28S/18S rRNA brightness ratio ≈ 2:1), and purity/concentration was quantified with a microvolume spectrophotometer ($OD_{260/280}$ = 1.8–2.1, $OD_{260/230} \geq 1.8$). Only qualified RNA was used for reverse transcription.

2.3.6.2 cDNA Synthesis

First-strand cDNA was synthesized using a reverse transcription kit with genomic DNA (gDNA) removal function. For gDNA elimination, 1–2 μg of total RNA was mixed with 3 μL 5× gDNA Wiper Buffer, adjusted to 15 μL with RNase-free water, and incubated at 42°C for 2 min. Then 5 μL 4× reverse transcription supermix was added to a final volume of 20 μL, with reactions performed at 37°C for 15 min, followed by enzyme inactivation at 85°C for 5 s. The resulting cDNA was stored at -80°C until use.

2.3.6.3 Primer Design and Amplification Efficiency Validation

Primers for target and reference genes were designed using Primer Premier 5.0 based on coding sequences (CDS) from the NCBI database, with parameters: Tm 58–62°C, amplicon length 150–250 bp, GC content 40–60%, and exon-spanning design to avoid gDNA amplification. Primer specificity was confirmed via gradient PCR and agarose gel electrophoresis. Amplification efficiency was validated via 5-point 10-fold serial dilution standard curves; all primer pairs used had amplification efficiencies of 90–110% with a linear correlation coefficient $R^2 \geq 0.99$.

2.3.6.4 qRT-PCR Amplification and Data Analysis

qRT-PCR was performed on the Applied Biosystems 7500 Real-Time PCR System using a SYBR Green fluorescent quantification kit. The 20 μL reaction system contained 1 μL cDNA template, 0.4 μL each of forward and reverse primers (10 μM), 10 μL 2× SYBR Master Mix, and 8.2 μL RNase-free water. The thermal program was set as: 95°C pre-denaturation for 30 s; 40 cycles of 95°C denaturation for 10 s, 60°C annealing/extension with fluorescence acquisition for 30 s; followed by melting curve analysis (60°C to 95°C) to confirm single specific amplification products. The rice housekeeping gene OsActin was used as the reference gene for data normalization. Four biological replicates and three technical replicates were set for each sample, and relative gene expression was calculated using the $2^{-\Delta\Delta Ct}$ method [21].

Table S1 Information on key genes in photosynthesis, ROS, Na^+, and K^+ in response to combined heat and salt stresses

Gene Name	Description
APX2	L-ascorbate peroxidase 2, cytosolic
SODCC1	Cu^{2+}/Zn^{2+} Superoxide dismutase 1, cytosolic, chloroplastic
APX1	cytosolic ascorbate peroxidase 1
psbo	33kDa precursor protein of oxygen-evolving complex
RbcS2	ribulose bisphosphate carboxylase small chain 2A
RbcS3	ribulose bisphosphate carboxylase small chain 3B
OsSOS1	Drives active Na^+ efflux
OsSOS2	while protecting K^+ uptake,
OsSOS3	centrally maintaining intracellular K^+/Na^+ balance.
OsHKT1;3	Selectively transports Na^+ to regulate its distribution among root,
OsHKT1;5	stem, and leaf<br>minimizing Na^+ toxicity in critical tissues.
OsNHX4	Sequesters excess cytosolic Na^+ into vacuoles by active pumping.
OsNHX5	

Table S2 qRT-PCR primer sequence information

Gene	Forward Primer Sequence（5'-3'）	Reverse Primer Sequence（5'-3'）
PsbO	CTCTACCGGCTACGACAACG	CTCTGGCTTGCTCTTGGTGA
OsAPX2	TCCTACGCCGACTTCTACCA	ACCCATCTGCGCAGAAAAGA
OssodCc1	AAGTGTCTCTGGGCTCAAGC	CTCATCTTGTGGTGCCCCAT
OsAPX1	ACGCCGATTTCTACCAGCTT	GCCTTAGGTGGTCAGAACCC
OsRBCS2	GCAGATCGAGTACCTGCTCC	CAGCTTCCACATGGTCCAGT
OsRBCS3	CCTATCGTACCTGCCACCAC	CAGCTTCCACATGGTCCAGT
OsSOS1	TCTGCAAAGGAGTGCGTCAT	TCATGCTCCCGTACATGCTC
OsSOS2	TTGGACTTAGCACTTTGGCCC	CTCAAAAGGAAGGTATCCAGCC
OsSOS3	TTCGCAGACAGGGTGTTTGA	TGCCAAGACCATCTCCCTGA
OsHKT1;3	GAACTCCGCATGGAAAATGGAG	CGATGATCCCAATGCCTTTTCT
OsHKT1;5	CGTCGAGGTTATCAGTGCGT	GCTTCCCTTGTTTGCTCCAC
OsNHX4	GTTGCTACTGGCTCTTCCCAA	ACTCCTTCCCCAAACACCAAA
OsNHX5	TGTTCTAAATGATGCGATGGCG	ACACCACAATGCCAAAGGTC

Table S3. Significance of rice physiological parameters under salt and heat stress

Parameter	Salt	Heat	Salt+Heat
H_2O_2 at post-heat stress	**	**	*
O_2^- at post-heat stress	**	**	*
MDA at post-heat stress	**	**	ns
SOD at post-heat stress	**	**	**
CAT at post-heat stress	**	**	ns
POD at post-heat stress	**	**	*
NR at post-heat stress	**	**	ns
GS at post-heat stress	**	**	**
GOGAT at post-heat stress	**	ns	ns
Pn at post-heat stress	**	**	**
Tr at post-heat stress	**	**	ns
Ci at post-heat stress	*	ns	ns
G_s at post-heat stress	**	**	ns
K^+ at post-heat stress	**	**	ns
Na^+ at post-heat stress	**	**	**
K^+/Na^+ at post-heat stress	**	**	*
SS at post-heat stress	**	**	ns
SPS at post-heat stress	**	**	ns
SSS at post-heat stress	**	**	ns
H_2O_2 at heading	**	**	ns
O_2^- at heading	**	**	ns
MDA at heading	**	*	ns
SOD at heading	**	**	ns
POD at heading	**	**	ns
CAT at heading	**	**	*
NR at heading	**	**	*
GS at heading	**	**	ns
GOGAT at heading	**	**	ns
Pn at heading	**	**	**
E at heading	**	ns	ns
Ci at heading	**	ns	*
G_s at heading	**	*	**
SS at post-heat stress	**	**	**
SPS at post-heat stress	**	**	**
SSS at post-heat stress	**	**	ns
OsAPX2 at post-heat stress	**	**	**
OsSODCC1 at post-heat stress	**	**	**
OsAPX1 at post-heat stress	**	**	**
Ospsbo at post-heat stress	**	**	*
OsRbcS2 at post-heat stress	**	**	**
OsRbcS3 at post-heat stress	**	**	**
OsSOS1 at post-heat stress	**	**	**

OsSOS2 at post-heat stress	**	**	**
OsSOS3 at post-heat stress	**	**	**
OsHKT13 at post-heat stress	**	**	**
OsHKT15 at post-heat stress	**	**	**
OsNHX4 at post-heat stress	**	**	**
OsNHX5 at post-heat stress	**	ns	**
1000-grain weight	**	**	*
Seed-setting rate	**	**	*
Grains per panicle	**	**	ns
Productive panicle per plant	**	**	*
Panicle development	**	**	**
Yield	**	**	**
Aboveground biomass at heading	**	ns	ns
Aboveground biomass at maturity	**	**	**
Leaf biomass at heading	**	**	ns
Leaf biomass at maturity	ns	**	*
Stem sheath biomass at heading	**	ns	**
Stem sheath biomass at maturity	**	**	**
Leaf aboveground biomass translocation	**	ns	ns
Stem sheath aboveground biomass translocation	**	**	**
Leaf N accumulation at heading	ns	**	ns
Leaf N accumulation at maturity	*	ns	ns
Stem sheath accumulation at heading	**	ns	ns
Stem sheath accumulation at maturity	ns	**	ns
Total N accumulation at heading	**	ns	ns
Total N accumulation at maturity	**	**	*
Leaf N translocation	ns	**	ns
Stem sheath N translocation	**	ns	ns
NSC accumulation at heading	**	**	**
NSC accumulation at maturity	**	**	**
NSC translocation	**	**	ns
NSC translocation	**	**	ns

Note: * indicates a significant difference compared with the non-stressed control (CK) at $P < 0.05$; ** indicates an extremely significant difference compared with CK at $P < 0.01$; ns indicates no significant difference.

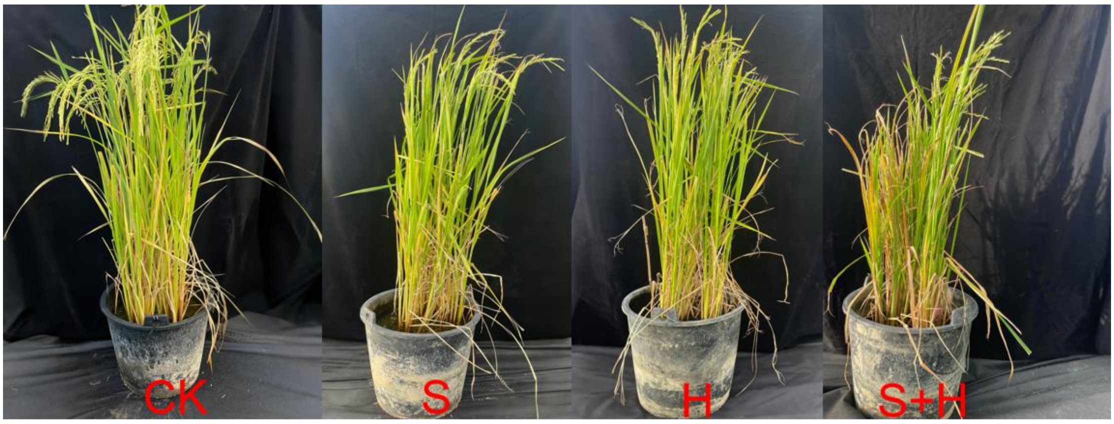

Figure S1 Photos of rice at the heading stage

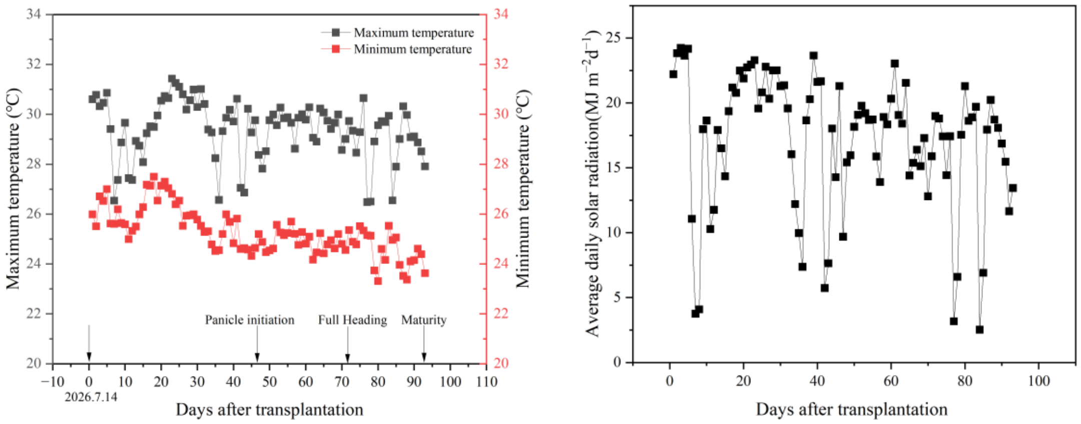

Figure S2 The maximum and minimum temperature and average daily solar radiation during the experiment.

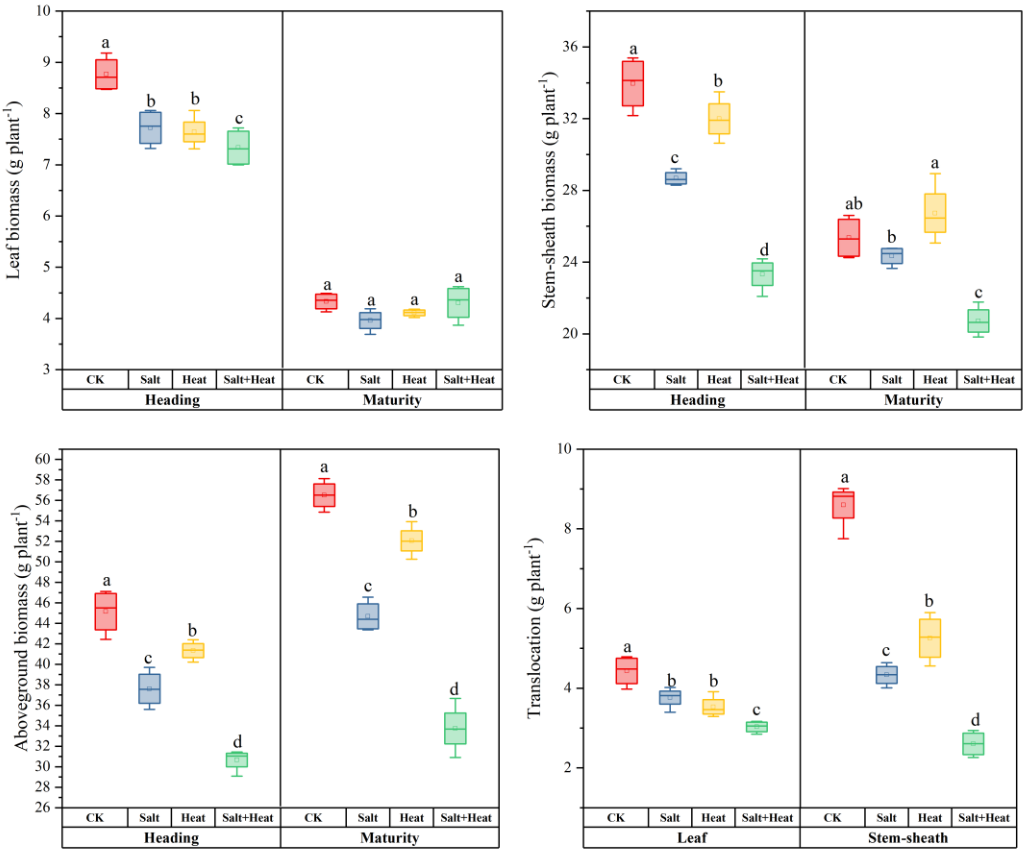

Figure S3. Aboveground biomass and translocation at the heading and maturity stage. (a) Leaf biomass; (b) Stem sheath biomass; (c) Aboveground biomass; (d) Aboveground biomass translocation

Data are mean ± SE (n = 4). Different lowercase letters indicate significant differences among treatments at $p < 0.05$ level by Tukey's HSD test.
